# The Microbiota of the Human Mammary Ecosystem

**DOI:** 10.3389/fcimb.2020.586667

**Published:** 2020-11-20

**Authors:** Leónides Fernández, Pia S. Pannaraj, Samuli Rautava, Juan M. Rodríguez

**Affiliations:** ^1^ Department of Galenic Pharmacy and Food Technology, Complutense University of Madrid, Madrid, Spain; ^2^ Department of Pediatrics and Molecular Microbiology and Immunology, Keck School of Medicine and Children’s Hospital, Los Angeles, CA, United States; ^3^ University of Helsinki and Helsinki University Hospital, New Children’s Hospital, Pediatric Research Center, Helsinki, Finland; ^4^ Department of Nutrition and Food Science, Complutense University of Madrid, Madrid, Spain

**Keywords:** human milk, microbiota, microbiome, vertical transfer, biological functions, mastitis, breast tissue, breast cancer

## Abstract

Human milk contains a dynamic and complex site-specific microbiome, which is not assembled in an aleatory way, formed by organized microbial consortia and networks. Presence of some genera, such as *Staphylococcus, Streptococcus, Corynebacterium, Cutibacterium* (formerly known as *Propionibacterium*), *Lactobacillus*, *Lactococcus* and *Bifidobacterium*, has been detected by both culture-dependent and culture-independent approaches. DNA from some gut-associated strict anaerobes has also been repeatedly found and some studies have revealed the presence of cells and/or nucleic acids from viruses, archaea, fungi and protozoa in human milk. Colostrum and milk microbes are transmitted to the infant and, therefore, they are among the first colonizers of the human gut. Still, the significance of human milk microbes in infant gut colonization remains an open question. Clinical studies trying to elucidate the question are confounded by the profound impact of non-microbial human milk components to intestinal microecology. Modifications in the microbiota of human milk may have biological consequences for infant colonization, metabolism, immune and neuroendocrine development, and for mammary health. However, the factors driving differences in the composition of the human milk microbiome remain poorly known. In addition to colostrum and milk, breast tissue in lactating and non-lactating women may also contain a microbiota, with implications in the pathogenesis of breast cancer and in some of the adverse outcomes associated with breast implants. This and other open issues, such as the origin of the human milk microbiome, and the current limitations and future prospects are addressed in this review.

## Introduction

Historically human milk was considered sterile under physiological conditions and, therefore, the presence of microbes was considered either as an infection or as a contamination. The mammary glands are made up of a moist intra-mammary mucosal ecosystem which, during late pregnancy and throughout the lactation period, becomes an ideal environment for bacterial growth due to the availability of a wide range of nutrients, and the optimum temperature for many microbes. In addition, the extremely complex duct system may favor the growth and spreading of biofilm-forming bacteria, a property that is common among *Staphylococcus* strains isolated from human milk (Delgado et al., 2009; Delgado et al., 2011; Begović et al., 2013). In addition, the mammary ecosystem is exposed to the external environment (via the ducts/nipple) and, also, to the internal environment since tight junctions remain open for a few days after birth. Since the first culture-dependent and -independent reports of the human milk microbiome (Heikkilä and Saris, 2003; Martin et al., 2003), all published studies have provided evidence of the presence of bacteria or bacterial DNA in milk collected from healthy women under hygienic conditions. In contrast, no modern study has found evidence of their absence.

## The Microbiota of Human Milk: Historical Perspective

**Figure 1:**
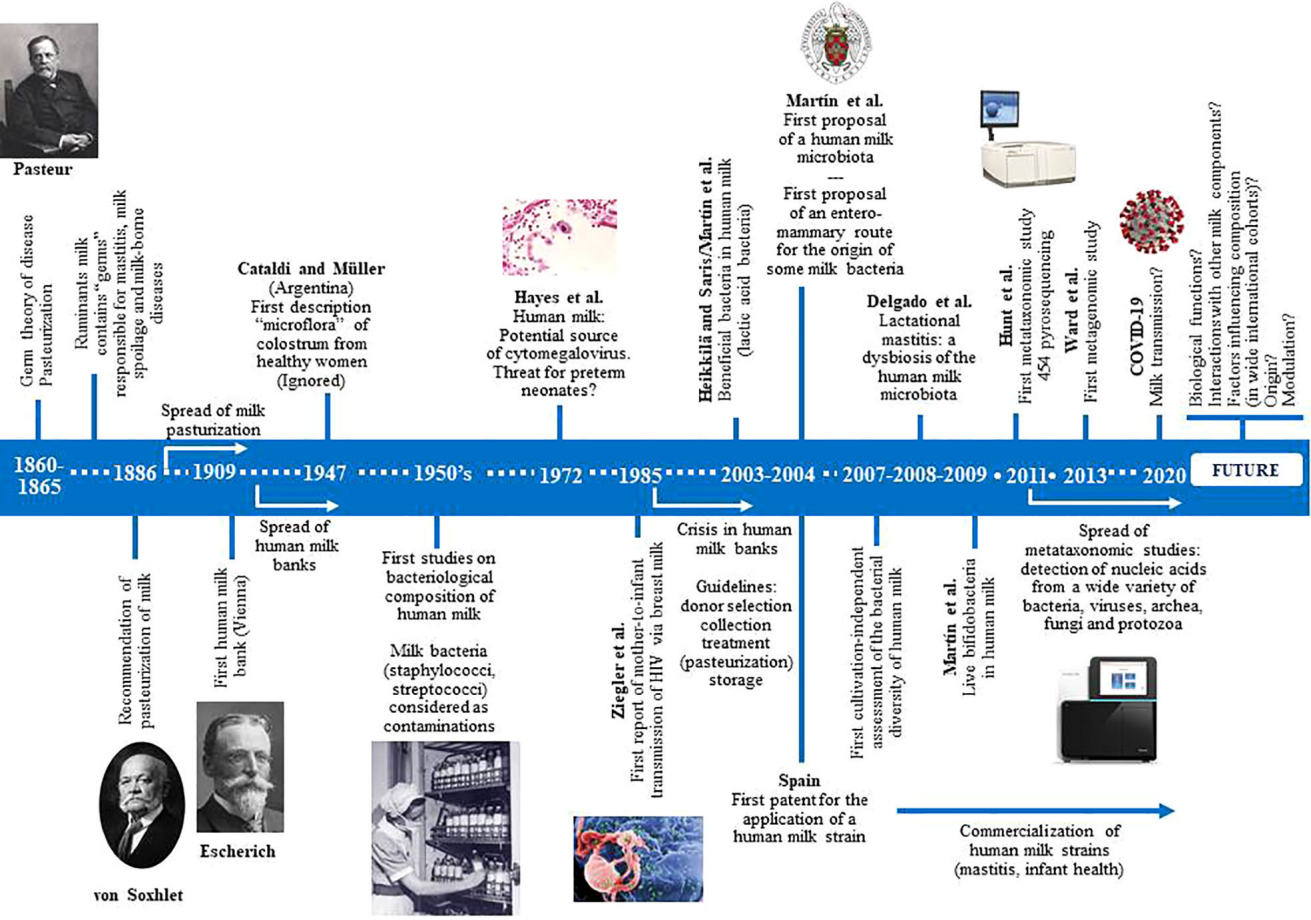
Historical perspectives of the main milestones in research focused in human milk microbes.

Knowledge of the bacterial content of fresh mammalian milk is as old as Pasteur’s “germ” theory (**Figure 1**). Numerous studies, dating back more than a century ago, demonstrated that bacteria are common in milk of healthy ruminants. However, milk microbes were traditionally viewed from three perspectives: (a) as a potential cause of mastitis, leading to economic losses in farms (Macfadyen, 1891; French, 1903; Jones, 1918); (b) as a potential cause of milk spoilage (Hall and Trout, 1968); or (c) as a potential threat to human health because of the potential transfer of pathogenic microbes, including those with a zoonotic origin and those arising from a non-hygienic handling (Anonymous, 1889; Park, 1901). Many milk-borne diseases, including tuberculosis, brucellosis, typhoid fever, diphtheria or scarlet fever, had been recognized before 1900 (Holsinger et al., 1997), and that it is estimated that approximately 65,000 people died of milk-borne tuberculosis in England alone between 1912 and 1937 (Wilson, 1943). More than half a century later (mid-1950s), the first studies dealing with the bacteriological composition of human milk were published (Lindemann and Rupp, 1954; Novel and Pongratz, 1954; Lindemann, 1955; Mocquot, 1955; Meyer and Potel, 1956; Sager, 1956; Mossel and Weijers, 1957; Lindemann, 1958). Most of them reported an unexpected fact: the abundant and widespread bacterial “contamination” of milk donated to human milk banks. Since these institutions were rapidly spreading in Western countries, the number of studies proposing either bacterial criteria for acceptance of donor milk, or collection, storage and processing procedures to reduce the milk bacterial load, also increased rapidly from the 1950s to the early 1980s (Bachmann and Pulverer, 1962; Krieg, 1966; Liebhaber et al., 1978; Carroll et al., 1979; Davidson et al., 1979; Eidelman and Szilagyi, 1979; Jones et al., 1979; Lucas and Roberts, 1979; West et al., 1979; Björkstén et al., 1980; Carroll et al., 1980).

In parallel, a number of studies warned about the presence of some potential pathogenic bacteria among such “contaminants”, which posed a risk for infant health. They included mainly *Staphylococcus aureus* but, also, *Streptococcus pyogenes*, *Streptococcus agalactiae* and some enterobacteria (*Salmonella* spp., *Klebsiella pneumoniae*) (Dubois, 1954; Cayla et al., 1955; Rantasalo and Kauppinen, 1959; Foster and Harris, 1960; Ottenheimer et al., 1961; Dluzniewska, 1966; Burbianka and Dluzniewska, 1971; Fleischrocker et al., 1972; Kenny, 1977; Ryder et al., 1977; Schreiner et al., 1977; Lucas and Roberts, 1978; Williamson et al., 1978; Donowitz et al., 1981; Cooke et al., 1987; Lemoine, 1987). Finally, a few studies were focused on the relationship between milk bacteria and mastitis (Dorr and Sittel, 1953; Cherkasskaia et al., 1980; Thomsen, 1982).

In 1985, the first report of a case of presumed mother-to-infant transmission of human immunodeficiency virus (HIV) *via* milk was published (Ziegler et al., 1985). In the following years, fear of HIV transmission led human milk banks to a strong crisis and to the closing of many of them because of the financial burden of serological testing of donors (Haiden and Ziegler, 2016). In addition, a few years earlier, human milk had also been recognized as a source of cytomegalovirus (CMV) (Hayes et al., 1972). This worsened the situation since most banked milk was intended for preterm neonates, a population where CMV may be particularly harmful, and no routine procedure for CMV screening was available at that time. As a result, most microbial studies of human milk published in the second half of the 80’s and throughout the 90’s were related to its role as a vehicle of HIV and/or CMV, and to the search for the best methods for donors screening and for viral inactivation (Stagno et al., 1980; Friis and Andersen, 1982; Cheeseman and McGraw, 1983; Dworsky et al., 1983; Boyes, 1987; Anonymous, 1988). At this stage, presence of any kind of microbe in milk was generally seen as a potential threat for infant health.

This disease-centric view of milk changed in 2003 following the publication of two articles that described the presence of lactic acid bacteria in human milk (Heikkilä and Saris, 2003; Martin et al., 2003). Such bacteria were generally recognized as safe and beneficial for infant health. One year later, it was proposed that human milk contains its own site-specific microbiota (Martín et al., 2004). Soon, it was found that some strains isolated from human milk displayed a wide array of probiotic traits (Beasley and Saris, 2004; Martín et al., 2005; Martín et al., 2006; Olivares et al., 2006a). In fact, the first description of the presence of lactobacilli (*Lactobacillus acidophilus*) and bifidobacteria (then the so-called *Lactobacillus bifidus*) in colostrum from healthy women was made in 1947 (Cataldi and Müller, 1947); however, such work was (and still is) ignored by the medical and scientific community, likely due to the fact that it was published in an Argentine journal with no diffusion outside of Spanish-talking countries.

Presence of microorganisms in human milk is no longer valid as a predictor of the risk of infant infection (Boer et al., 1981; Schanler et al., 2011; Zimmermann et al., 2017), despite the fact that additional cases of human milk-related infant infections have been reported (Qutaishat et al., 2003; Kayıran et al., 2014; Weems et al., 2015).

## The Composition of the Human Milk Microbiota

From 2003, the study of the human milk microbiota has attracted the interest of many research groups worldwide (Fernandez et al., 2013; Jost et al., 2015; Ojo-Okunola et al., 2018), enabling the detection of approximately 200 different bacterial, archeal and fungal species from more than 50 different genera (Fernandez et al., 2013), including new species (Martín et al., 2011), and new genera, including *Lactomassilus*, *Lactimicrobium*, *Anaerolactibacter*, *Galactobacillus*, and *Acidipropionibacterium* (Togo et al., 2017; Togo et al., 2019a).

Culture-based methods have revealed that some species of the genera *Staphylococcus* (*Staphylococcus epidermidis* and other coagulase-negative species [CNS]), *Streptococcus* (*S. salivarius*, *S. mitis* and other species of the mitis group), *Corynebacterium*, *Cutibacterium* and other taxonomically-related Gram-positive bacteria are usually the dominant cultivable bacteria in samples of milk from healthy women (Jiménez et al., 2008a; Jiménez et al., 2008b; Solís et al., 2010; Schanler et al., 2011; Ding et al., 2019). Less frequently, lactic acid bacteria (*Lactococcus*, *Enterococcus*, *Lactobacillus*, *Leuconostoc*, and *Weissella*) and bifidobacteria are isolated from this biological fluid (Martin et al., 2003; Martín et al., 2006; Abrahamsson et al., 2009; Martín et al., 2009; Solís et al., 2010; Arboleya et al., 2011; Makino et al., 2015; Murphy et al., 2017). Some *Lactobacillus* (*L. salivarius*, *L. reuteri, L. gasseri*, *L. fermentum*) and *Bifidobacterium* (*B. breve* and *B. longum*) species have received particular interest because of the potential of the strains belonging to such species to be employed as probiotics. It must be highlighted that the different species of the genera *Lactobacillus* and *Leuconostoc* have been recently reclassified into 25 different genera (Zheng et al., 2020).

Under physiological conditions, milk bacterial concentrations may range from <1 to 4 log_10_ colony-forming units (cfu)/mL if the samples are obtained by either manual expression or through sterile pumps following hygienic practices (Espinosa-Martos et al., 2016). In contrast, bacterial concentrations can rise up to 6 log_10_ cfu/mL, or even higher, in mastitis cases (Fernández et al., 2014) or if milk is collected through the use of non-sterile pumps (Boo et al., 2001; Brown et al., 2005; Marín et al., 2009; Jiménez et al., 2017). At present, cell count methods, from classic counting chambers to flow cytometry, are the best methods for an accurate quantification of (live) bacterial cells in milk while quantitative PCR methods usually lead to an overestimation due to the presence of dead bacterial cells, exosomes and/or free bacterial DNA but, also, to mispriming with human DNA (Walker et al., 2020), and copy number bias (Větrovský and Baldrian, 2013; Louca et al., 2018).

Introduction of new media, supplements and incubation conditions have allowed the isolation of bacteria that were previously unnoticed. However, recent developments in culturomics have revealed that many bacteria previously regarded as non-cultivable can now be isolated from complex ecosystems when a proper combination of culture conditions is provided (Lagier et al., 2012; Lagier et al., 2015; Lau et al., 2016; Lagier et al., 2018; Schwab et al., 2019). These new methodologies have served as critical tests to validate data derived from metagenomic studies regarding the gut ecosystem (Lau et al., 2016). Culture-based techniques enable the isolation, preservation and characterization of strains (Bäckhed et al., 2005; Lara-Villoslada et al., 2007; Jiménez et al., 2008b; Delgado et al., 2009; Jiménez et al., 2010a; Jiménez et al., 2010b; Arboleya et al., 2011; Delgado et al., 2011; Gueimonde et al., 2012; Jiménez et al., 2012; Langa et al., 2012; Martín et al., 2012a; Martín et al., 2013; Cárdenas et al., 2014; Cárdenas et al., 2015).

**Figure 2:**
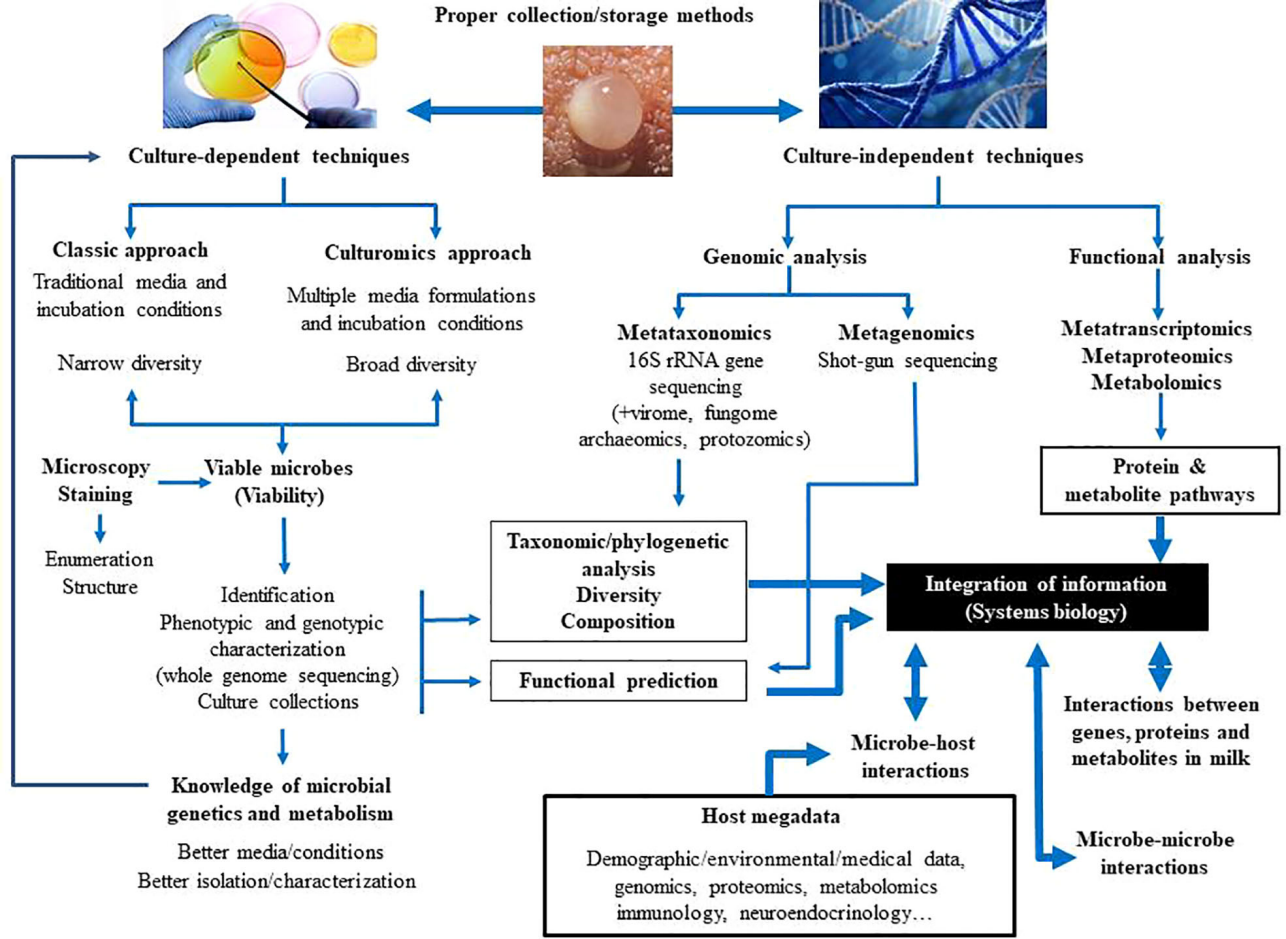
Current and future approaches to study the composition and functions of the human milk microbiota.

In relation to the milk microbiome, the use of first generation of culture-independent techniques, including PCR, combined or not with creation of bacterial gene libraries, denaturing gradient gel electrophoresis (DGGE), and temperature gradient gel electrophoresis (TGGE), allowed a better knowledge of the milk bacterial populations (Gueimonde et al., 2007; Martín et al., 2007a; Martín et al., 2007b; Delgado et al., 2008; Collado et al., 2009). Nowadays, they have been replaced by high-throughput Next Generation Sequencing (NGS) techniques, including metataxonomics (16S rRNA amplicon analysis) and metagenomics (total DNA sequencing). Sequencing of bacterial 16S rRNA genes has revealed that milk contains DNA of diverse microbial groups that were previously undetected with conventional culture-based techniques (Hunt et al., 2011; Cabrera-Rubio et al., 2012; Jost et al., 2013; Jost et al., 2014; Cabrera-Rubio et al., 2016; Boix-Amorós et al., 2017; Pannaraj et al., 2017). Although results from such studies do not provide evidence for viability, they have been useful to highlight the complexity of the milk microbiota and its role in modulating mammary homeostasis and gut colonization during early life. Shotgun sequencing of milk microbial DNA has been rarely performed to date (Ward et al., 2013; Jiménez et al., 2015), despite the fact that it can reveal the presence and potential functions of neglected members of this microbiota (archaea, viruses, fungi, protozoa). In addition, although DNA-based studies cannot demonstrate function, they can imply functional capacity based on the genes present in the samples. Comprehensive metagenomic, metatranscriptomic and metabolomic investigations are required for a holistic understanding of genetic diversity and functionality within the milk ecosystem (**Figure 2**).

Human milk microbiota is not aleatory assembled and contains organized bacterial consortia and networks (Ma et al., 2015; Drago et al., 2017). The milk microbiota of a woman is very stable throughout the lactation period, particularly in relation to the most abundant genera (Hunt et al., 2011; Williams et al., 2017a). Culture-independent studies have shown a wide diversity of bacterial signatures belonging to more than 800 different bacterial species, mainly from four major phyla (*Firmicutes*, *Actinobacteria*, *Bacteroidetes*, and *Proteobacteria*) (Togo et al., 2019b), including all the cultivable species (Gueimonde et al., 2007; Martín et al., 2007a; Martín et al., 2007b; Delgado et al., 2008; Collado et al., 2009; Hunt et al., 2011; Cabrera-Rubio et al., 2012; Jost et al., 2013; Ward et al., 2013; Jost et al., 2014; Jiménez et al., 2015; Boix-Amorós et al., 2016; Cabrera-Rubio et al., 2016; Sakwinska et al., 2016; Chen et al., 2018; Simpson et al., 2018; Tuominen et al., 2018; Ding et al., 2019; Lackey et al., 2019). Additionally, DNA from some gut-associated strict anaerobes (*Faecalibacterium, Bacteroides*, *Clostridium*, *Blautia, Coprococcus*, *Ruminococcus, Roseburia*, *Eubacterium, Veillonella*, and others) has also been repeatedly detected (Cabrera-Rubio et al., 2012; Jost et al., 2013; Jost et al., 2014; Jiménez et al., 2015; Gómez-Gallego et al., 2016).

Different studies have also reported the frequent detection of DNA sequences from soil- and water-related bacteria, including *Bradyrhizobium*, *Novosphingobium*, *Methylobacterium*, *Pseudomonas*, *Sphingobium*, *Sphingopyxis*, *Stenotrophomonas*, *Sphingomonas* or *Xanthomonas*, in human milk (Hunt et al., 2011; Cabrera-Rubio et al., 2012; Urbaniak et al., 2014a; Urbaniak et al., 2014b; Li et al., 2017). It is possible that a high proportion of such sequences are the result of technical artifacts. DNA from the bacterial genera cited above is generally present in molecular biology reagents, solutions and kits (Grahn et al., 2003; Mühl et al., 2010; Salter et al., 2014; Lauder et al., 2016). This represents a major challenge assessing the composition of microbiotas characterized by a low microbial biomass, as is the case of the human milk microbiota in healthy women. Upon amplification, contaminating DNA may overcome the low amount of starting material in the biological sample and lead to incorrect results (Laurence et al., 2014). Sequences belonging to such genera (e.g., *Pseudomonas*) may be so abundant that they can be wrongly included into the milk core microbiome. There are several measures that can be implemented to minimize this problem, such as sequencing of negative (blank) controls and contaminant removal procedures (Salter et al., 2014; Hornung et al., 2019; Karstens et al., 2019; Stinson et al., 2019), while providing microbial DNA-free sampling containers and molecular reagents is still a pending challenge for companies working in this field.

Differences in the techniques and procedures employed in different studies may account, at least partly, for conflicting results regarding the frequency and abundance of sequences belonging to *Lactobacillus*, *Bifidobacterium* and strict anaerobes in human milk (Lagier et al., 2012). These kind of controversies have also happened in relation to the microbiota of the infant gut (Palmer et al., 2007; Turroni et al., 2012). Other limitations and biases of molecular techniques include the lack of discrimination between live or dead organisms when techniques compatible with viability assessments are not selected (Emerson et al., 2017), and the over- or underestimation of some microbial groups because of the composition of their plasmatic membranes, outer membranes or cell walls, methods used for extraction of nucleic acids, copy number of the target gene, the specific 16S rRNA region(s) targeted by the selected primers, and the pipelines used for the bioinformatic analysis (McGuire and McGuire, 2015; Gómez-Gallego et al., 2016; McGuire and McGuire, 2017).

Some studies have revealed the presence of cells, DNA and/or RNA from viruses, archea, fungi and protozoa in human milk (Jiménez et al., 2015; Pannaraj et al., 2018; Boix-Amorós et al., 2019). Although some pathogenic viruses, such as HIV, CMV, Ebola and Zika viruses may be found in human milk (Kourtis et al., 2003; Van de Perre et al., 2012; Bardanzellu et al., 2019; Sampieri and Montero, 2019; Ververs and Arya, 2019), other viruses, including bacteriophages, are also present (Mohandas and Pannaraj, 2020). The human milk virome is distinct from other body anatomical sites and includes eukaryotic viruses, bacteriophages, and viral elements integrated in the host chromosomes (Duranti et al., 2017; Pannaraj et al., 2018). The most abundant eukaryotic viruses belong to the families *Herpesviridae, Poxviridae, Mimiviridae* and *Iridoviridae.* Bacteriophages comprise 95% of the human milk viruses and have the ability of modulating the bacterial ecology by killing specific bacteria or by supplying them with additional gene functions (Pannaraj et al., 2018). Human endogenous retroviruses, accounting for 0.06 to 3.63% of all reads, have also been identified in milk samples (Jiménez et al., 2015). Both pathogenic and non-pathogenic viruses can be vertically transferred from mother to infant (Kuehn et al., 2013; Lugli et al., 2016; Duranti et al., 2017; Blohm et al., 2018; Pannaraj et al., 2018).

Some studies have identified archaeal sequences in human milk (Ward et al., 2013; Jiménez et al., 2015; Togo et al., 2019c). In addition, Togo et al. (2019b) were able to isolate *Methanobrevibacter smithii* from 3 colostrum and 5 milk samples out of a total of 20 samples while *Methanobrevibacter oralis* was cultured from one milk sample. Methanogenic archaea have remained largely underestimated in human microbiome studies due to technical difificulties in their assessment (Bang and Schmitz, 2015). However, they are particularly adapted to the human gut, participating actively in metabolism and health through methanogenesis (Samuel et al., 2007; Dridi et al., 2009). Presence of *M.* *smithii* in the human gut is a feature of healthy lean adults while there is a depletion of this species in obese adults (Le Chatelier et al., 2013; Goodrich et al., 2014). In this context, *M*. *smithii* was less frequently detected by either culture or PCR in the milk samples obtained from obese mothers in the study of Togo et al. (2019c). The human gastrointestinal tract is colonized by *M*. *smithii* early in life (Grine et al., 2017), and colostrum and milk may represent relevant sources of methanogenic archaea.

In relation to fungi, a metagenomic analysis detected fungal-related sequences in 17 out of the 20 milk samples included in the study (Jiménez et al., 2015). More specifically, the reads belonged to the phyla *Basidiomycota* and *Ascomycota*, and to the species *Calocera cornea*, *Candida dubliniensis*, *Guepiniopsis buccina*, *Malassezia globosa*, *Malassezia restricta*, *Podospora anserina*, *Sordaria macrospora*, *Talaromyces stipitatus*, and *Yarrowia lipolytica*, with *M. globosa* being the most widespread species (Jiménez et al., 2015). Boix-Amorós et al. (2017) could visualize and isolate yeasts from 17 out of 41 milk samples from healthy women, and most of the isolates belonged to the species *Candida parapsilosis* and *Rhodotorula mucilaginosa.* Later, the same group analyzed 80 milk samples from women of 4 different countries by sequencing of the ITS1 region of the fungal rDNA gene, and found that *Malassezia* and *Davidiella* were the most prevalent genera independenty of the country, while delivery mode and geographic location were associated with shifts in the milk mycobiome composition (Boix-Amorós et al., 2017). Sequencing of the ITS2 region allowed the identification of *Candida* and *Saccharomyces* sequences in 6 milk samples from women whose infant stayed in a neonatal intensive care unit (NICU) althouh sequences of the same genera were also identified in samples from the NICU environment (Heisel et al., 2019). Overall, these studied suggest that milk may be a source of fungi for the infant gut, thus contributing to the acquisition and development of the gut mycobiota. However, more studies are required to confirm this role, and to elucidate the potential interactions with other microbes. The significance of the protozoa (*Toxoplasma gondii*, *Giardia intestinalis*) detected in milk from some mothers remains unclear (Jiménez et al., 2015).

## Factors Affecting the Composition of the Human Milk Microbiota

**Figure 3:**
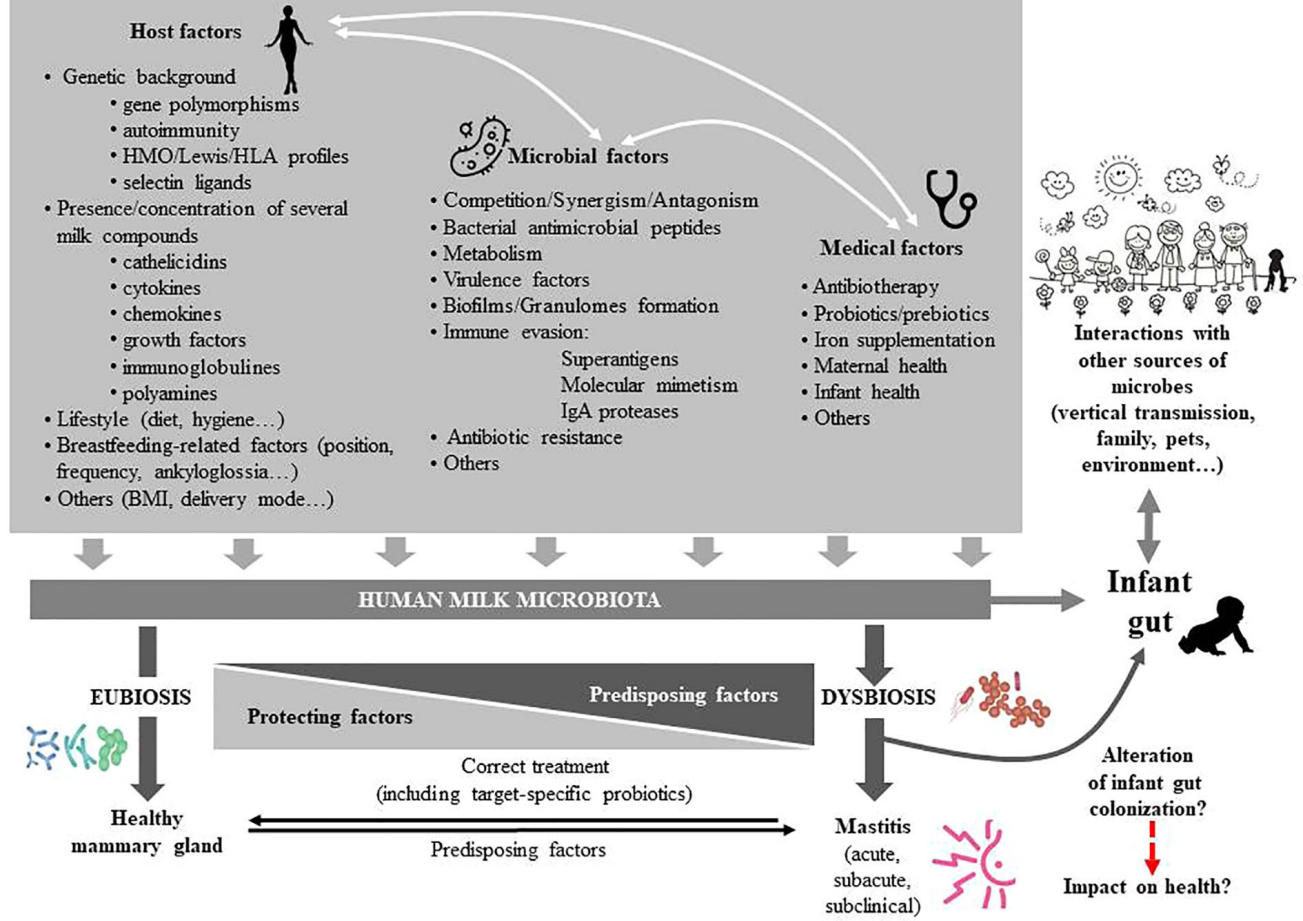
Factors affecting the composition of the human milk microbiota. HMO, human milk oligosaccharides; HLA, human leukocyte antigen; BMI, body mass index; IgA, immunoglobulin A.

The milk microbiome is characterized by a certain degree of interindividual variability (Martín et al., 2007a; Martín et al., 2007b; Hunt et al., 2011; Boix-Amorós et al., 2016; Cabrera-Rubio et al., 2016; Avershina et al., 2018; Chen et al., 2018). Modifications in its composition may have biological implications for infant colonization, metabolism, immune and neuroendocrine development and for mammary health. However, the current knowledge about the impact of a wide variety of factors (genetic background, ethnicity, milk sampling, geographical location, circadian rhythm, maternal age, diet and body mass index [BMI], delivery mode, gestational age, therapies and food supplements, infant and maternal health status, and others) on human milk microbial communities is very limited (Fernández et al., 2014; Gómez-Gallego et al., 2016) (**Figure 3**).

Although some studies have tried to elucidate the influence of some of these factors, most of them involved a low number of samples/women and/or have relied on short amplicon sequencing, a technology which poor resolution at the lower taxonomical levels may mask differences or overinflate them; these limitations together with the fact that many factors with a potential impact on the composition of the milk microbiome may interact, makes it difficult to evaluate their true impact on the milk microbiome (LeMay-Nedjelski et al., 2018).

### Sample Collection

When milk is collected through the use of domestic (non-single use) milk pumps, a high concentration of yeasts and Gram-negative bacteria (*Enterobacteriaceae*, *Stenotrophomonas*, *Pseudomonas*) may arise from the water used to rinse the devices or as a consequence of unhygienic practices (Knoop et al., 1985; Moloney et al., 1987; Thompson et al., 1997; Boo et al., 2001; Brown et al., 2005; Marín et al., 2009; Jiménez et al., 2017); in fact, some cases of neonatal and infant sepsis and infections initially associated with human milk were the result of contamination of breast pumps (Gransden et al., 1986). A recent study comparing milk samples from mothers who regularly used pumps versus those who never used pumps showed that pumped milk was consistently related to an enrichment of potential pathogens compared to hand expressed milk (Moossavi et al., 2019a). This observation suggests that regular pump use may alter the milk microbiome over time. In fact, it has been shown that when sterile single-use pumps are used, no difference is detected between hand-expressed and pump-expressed samples, while the use of the mother’s own multi-use pump does result in a significant change in the apparent milk microbiome (Rodríguez-Cruz et al., 2020).

### Human Milk Oligosaccharides

Human milk oligosaccharides (HMOs) exert prebiotic effects on some bacteria that are frequently detected in human milk, including *Bifidobacterium* spp. and *Staphylococcus* *epidermidis* (Hunt et al., 2012; Thongaram et al., 2017). Milk oligosaccharides and microbes confer a highly personalized “symbiotic” complex which may be crucial for the development of the infant gut microbiota (Jost et al., 2015; Lewis et al., 2015; Wang et al., 2015; Williams et al., 2017a; Moossavi et al., 2019b). Positive correlations between HMO concentration and the abundance of *Staphylococcus* (Williams et al., 2017b), between *B. breve* and sialylated HMOs, between *B.* *longum* group and non-fucosylated/non-sialylated HMOs, between fucosylated HMOs and *Akkermansia* *muciniphila*, and between fucosylated/sialylated HMOs and *S.* *aureus* have been described (Aakko et al., 2017). A study found that the maternal secretor status was associated with the composition of the milk microbiota for the first 4 weeks after parturition (Cabrera-Rubio et al., 2019). Although no differences on diversity and richness were detected, *Lactobacillus* spp., *Enterococcus* spp., *Streptococcus* spp., and *Bifidobacterium* spp. were lower or less prevalent in non-secretor samples than in secretor samples (Cabrera-Rubio et al., 2019).

There are several mechanisms by which HMOs may modulate the human milk microbiota, including prebiotic, antimicrobial, or immunomodulatory properties (Ackerman et al., 2017; Ackerman et al., 2018; Triantis et al., 2018; Bode, 2020). The ability to assimilate, metabolize or use HMOs is conserved among the *Bifidobacterium* species that are most frequently detected in the infant gut and in human milk (Sela and Mills, 2010; Underwood et al., 2015), although the pathways vary among species and strains (Sakanaka et al., 2019). However, a recent study has shown that there is not a strict correlation between bifidobacterial populations in human milk and their ability to metabolize HMOs (Lugli et al., 2020). The exact growth-promoting effect of HMOs on *Staphylococcus* isolates from human milk remains unknown, since these bacteria do not metabolize these compounds (Hunt et al., 2012). Recently, novel pathways enabling the growth of *Roseburia* and *Eubacterium* (two butyrate-producing genera that have been repeteadly detected in human milk) on HMOs have been published (Pichler et al., 2020). More studies are required to elucidate how HMOs can contribute to shape the microbiota of milk.

### Other Human Milk Metabolites

The complexity and dynamic chemical composition of human milk represents a challenge for researchers (Ojo-Okunola et al., 2020). In addition to HMOs, human milk contains a plethora of metabolites produced by either human or bacterial cells that are relevant for mammary and infant health (Gay et al., 2018). Some of them have the potential to shape the composition of the human milk and/or the infant gut microbiotas because of their roles in changing environmental conditions that are relevant for bacterial growth, such as the redox potential or by other mechanisms that promote or inhibit the growth of certain species or strains. Up to 68 metabolites were identified in one study assessing the metabolic and microbiota profiles of milk from 79 women from Finland, Spain, South Africa, and China. Bacilli, Actinobacteria. and Proteobacteria were the bacterial groups displaying more positive or negative associations with milk metabolites (Gómez-Gallego et al., 2018).

Short chain fatty acids (SCFA), including butyrate, acetate, and propionate, are the result of bacterial metabolism in the maternal gut and may reach the mammary gland through the bloodstream. These metabolites are key players in human homeostasis because of their interactions with the microbiota, the immune system, and the neuroendocrine system, contributing to the immunological, metabolic, and neurological programming of the host (Stinson et al., 2020). In this context, the concentrations of acetate and butyrate in milk samples from atopic mothers are significantly lower than in samples from non-atopic mothers (Stinson et al., 2020). Levels of butyrate in milk have also been negatively associated with infant BMI and this fact may program healthy adiposity outcomes later in life (Prentice et al., 2019). Interestingly, administration of some *Lactobacillus* strains isolated from human milk (*Lactobacillus salivarius* CECT 5713, *Lactobacillus fermentum* CECT 5716) to infants has shown ability for increasing the fecal concentration of butyrate (Maldonado et al., 2010; Gil-Campos et al., 2012). Variations in the concentrations of some hormones present in human milk (leptin, insulin) have also been associated with changes in the composition of the infant gut microbiota, SCFA concentrations, and gut permeability (Lemas et al., 2016). Current knowledge on the interactions between the metabolome and the microbiota of human milk is scarce despite the fact that they may have a paramount relevance for infant and mammary outcomes. Integrative studies are required to address these complex interactions.

### Maternal Diet and Body Mass Index

Maternal diet is associated with the abundance of some bacterial genera (Williams et al., 2017a; Williams et al., 2017b). Protein intake is positively correlated with the abundance of *Gemella* while consumption of monounsaturated and saturated fatty acids is negatively related with the abundance of *Corynebacterium*; similarly, a negative correlation was found between Firmicutes and total carbohydrates, disaccharides, and lactose ingestion (Williams et al., 2017a). A previous study had revealed that monounsaturated fatty acids were positively associated with *Lactobacillus* genus, while the contrary was observed for Proteobacteria (Kumar et al., 2016).

An association between vitamin C intake during pregnancy and the bacterial diversity of milk has also been reported (Padilha et al., 2019a). The same study found a positive relationship between ingestion of some fatty acids (linoleic and polyunsaturated fatty acids) during lactation and the level of *Bifidobacterium* in milk.

In relation to BMI, prepregnancy BMI has been associated with a higher milk microbial diversity and a lower abundance of *Streptococcus* (Davé et al., 2016). Another study found that the abundance of *Granulicatella* in milk from overweight and obese mothers was higher than in milk from normoweight women  (Williams et al., 2017a). A recent study showed that the milk of mothers with a high postpartum BMI contained more *Staphylococcus* and less *Lactobacillus* and *Streptococcus* sequences than milk from normoweight mothers (Ding et al., 2019).

### Immune Cells

The lactating mammary gland is a component of the mucosal-associated immune system that displays unique features when compared with other mucosal sites. For example, it contains a low biomass microbiota in contrast to the high bacterial concentration that characterizes the intestinal or the upper respiratory tracts (Mestecky, 2020). In addition, the mammary gland is considered a relevant component of the infant immune system since this link enables maternal-infant immune dialogue (Brandtzaeg, 2010; Hassiotou and Geddes, 2015). It is long known that the gut microbiota is essential for programming the infant immune system and vice versa (Milani et al., 2017a). However, while the development of the immune system and the development of the microbiota are coordinated in the gut, they remain independently regulated in the breast (Niimi et al., 2018). Interactions between milk microbiota and the mammary immune system are poorly known despite the fact that maternal mononuclear cells can transport gut-derived bacteria and bacterial components to the breast during pregnancy and lactation (Perez et al., 2007). It has been speculated that this process facilitates the discrimination between pathogens and commensal microbes by the neonatal immune system (Perez et al., 2007).

Studies dealing with potential associations between the milk concentrations of bacteria and immune cells are scarce and have provided conflicting results from no correlation (Boix-Amorós et al., 2016) to a negative association between the relative abundance of *Serratia* and both the somatic cell count and the neutrophil concentration (Williams et al., 2017b). As in the case of HMOs and other milk metabolites, there is a need for integrative approaches to clarify the interactions between the immune system and the microbiota in the mammary ecosystem. Information on the potential roles of specific bacterial strains isolated from human milk on the infant immune system is provided in *Moving From Composition to Function*.

### Gestational Age, Mode of Delivery, and Postpartum Period

Controversial results have been obtained from studies comparing the human milk microbiome composition among women delivering preterm or term neonates. So, while Urbaniak et al. (2016a) did not detect differences in bacterial profiles between preterm and term births, Soeorg et al. (2017a) reported that milk of mothers of preterm infants had higher staphylococcal counts but lower species diversity compared with term mothers. The same authors found that the *S. epidermidis* strains that colonized the gastrointestinal tract and skin of preterm neonates were different to those found in milk while the opposite was observed in term neonates (Soeorg et al., 2017b). However, the gut of breast-fed preterm infants was gradually enriched with strains present in the milk of their mothers.

Concerning the delivery mode, Urbaniak et al. (2016a) found no significant differences in bacterial profiles between Cesarean section (either elective or non-elective) and vaginal deliveries, which is in contrast with the data provided by other authors (Khodayar-Pardo et al., 2014; Cabrera-Rubio et al., 2016; Kumar et al., 2016; Ding et al., 2019). Toscano et al. (2017) described many differences in the microbiome of colostrum depending on the mode of delivery. Compared to Cesarean section, vaginal delivery was associated with a lower abundance of *Staphylococcus*, *Pseudomonas*, and *Prevotella*. In addition, colostrum from women delivering by Cesarean section was associated with a higher number of bacterial hubs and was richer in environmental bacteria. In practice, it is difficult to separate the influence of the mode of delivery with other factors since, as an example, antibiotherapy administered per protocol to women delivering by Cesarean section may be responsable for some of the differences in the microbial population. A recent work reported that the effect of intrapartum antibiotic exposure was less decisive than that of delivery mode on the milk microbiome assessed one month after delivery (Hermansson et al., 2019).

In relation to the postpartum period, Hoashi et al. (2016) found changes in bacterial abundance and glycosylation patterns associated with the amount of time passed since delivery but only in milk from women who delivered vaginally. Drago et al. (2017) detected differences between the microbiomes of colostrum and milk since abundance of anaerobic intestinal bacteria was lower in colostrum than in mature milk. The permeability of the tight junctions in the mammary epithelium is greater in the first days after parturition and this fact determines major differences in the biochemical and immunological composition of colostrum with respect to mature milk. Such differences may be responsible for differences in the microbial composition of colostrum and mature milk. In addition, increasing exposures of the breast to the infant microbiota may also determine microbial changes over time.

Another study showed that the relative abundance of *Staphylococcus* in samples collected at 3 months postpartum was lower in comparison to samples collected at 10 days postpartum which, in turn, had a lower diversity of operational taxonomic units (OTUs) from the genera *Rothia*, *Veillonella* and *Granulicatella* (Simpson et al., 2018). Biagi et al. (2018) reported that the composition of the milk microbiota changes after the first infant latching, becoming more diverse and being dominated by oral microbes (*Rothia* and *Streptococcus*). In contrast, Li et al. (2017) found that neither the lactation stage nor the maternal BMI influenced the milk microbiota of Taiwanese and Chinese women.

### Geographical Location

Geographical location of the mother seems to exert a strong impact on the microbiota composition of human milk. A study including samples from mothers of Spain, Finland, South Africa and China found location-related differences in the relative abundance of Bacteroidetes, Actinobacteria and Proteobacteria (Kumar et al., 2016). Geographical differences in the microbiota of samples obtained from 133 mothers in seven regions of China and Taiwan have also been described (Li et al., 2017). Similarly, differences in the alpha diversity and *Lactobacillus* occurrence in milk were also observed depending on the region of China where the mothers were recruited (Ding et al., 2019). A study involving a higher number of samples from mothers living in Ethiopia, Gambia, US, Ghana, Kenya, Peru, Spain, and Sweden provided evidence of substantial variability within and across cohorts (Lackey et al., 2019).

There are several reasons that may explain why geographical location, even within the same country, might influence the bacterial composition of human milk. Ethnicity, genetic background, diet and climate vary across regions, but people with different ethnicity, genetic background, age, diet, housing, or contact with other people and animals usually coexist in the same village or town. The environmental microbiome (air, surfaces, water, plants, animals, food, waste, etc.) reflects the influences existing in a given place and might drive differences in the composition of the microbiome in any body site. Experiments to elucidate the impact of the geographical location on the milk microbiome are very challenging because of the high number of factors that may bias the interpretation of the data. They should have to take into account the factors cited above but also use identical protocols from sample collection, storage and shipping to data analysis, which is very difficult in practice.

### Maternal Treatments (Antibiotics, Probiotics, Prebiotics, and Chemotherapy) and Infections

Antibiotic administration is one of the main drivers of dysbiosis in mucosal surfaces and the lactating mammary gland does not seem to be an exception. Lactobacilli and bifidobacteria are more abundant in milk from women who are not treated with antibiotics during pregnancy, delivery or lactation (Soto et al., 2014). Similarly, the *Bifidobacterium* load in milk obtained in the first week after delivery was lower among women receiving antibiotic prophylaxis in comparison to the control group (Padilha et al., 2019b). It is interesting to note that significantly lower amounts of bifidobacteria have been found in milk of allergic mothers compared with non-allergic ones (Grönlund et al., 2007).

Soeorg et al. (2017a) found that the probability of finding *mecA*-positive CNS in milk increases if the mother is hospitalized during the first month after delivery or if the neonate received antibiotherapy or needed an arterial catheter. These authors suggested that the presence in milk of pathogenic *Staphylococcus* could be reduced by limiting the exposition of women to the hospital environment. Intrapartum antibiotic prophylaxis is associated with a higher presence of transmissible genes conferring resistance to antibiotics in milk, which are subsequently shared with their infants (Pärnänen et al., 2018)

In relation to probiotics, oral administration of the probiotic VSL#3 (a mix of 8 strains belonging to the species *B. breve*, *B. longum*, *L. acidophillus*, *Lactobacillus delbrueckii* subsp. *bulgaricus*, *Lactobacillus paracasei*, *Lactobacillus plantarum*, and *Streptococcus thermophilus*) to pregnant and lactating women led to higher concentrations of *Lactobacillus* and *Bifidobacterium* in colostrum and milk of those women who ingested the product when compared to the placebo group (Mastromarino et al., 2015). However, this effect was only seen among women with vaginal deliveries. In contrast, ingestion of milk containing *Bifidobacterium animalis* ssp. *lactis* Bb-12, *L. acidophilus* La-5 and *Lactobacillus rhamnosus* GG during late pregnancy and early lactation, did not modified significantly the composition of the milk microbiota (Simpson et al., 2018). Recently, Padilha et al. (2020) reported that the intake of prebiotics (fructooligosaccharides) affects the composition of the milk microbiota and found that this effect was influenced by the age of the mother.

Two studies studied the impact of chemotherapy employed during the treatment of Hodgkin’s lymphoma in the milk microbiome. The first study included milk samples obtained regularly during four months from a single treated woman and from 8 healthy lactating women (Urbaniak et al., 2014b). Chemotherapy led to significant changes in the milk bacterial composition, including an increase of *Xanthomonadaceae*, *Acinetobacter* and *Stenotrophomonas*, and a drastic reduction in the presence of *Eubacterium*, *Bifidobacterium*, *Cloacibacterium* and *Staphylococcus*. Changes in the metabolic profile of milk, characterized by a decrease of inositol and docosahexaenoic, were also observed (Urbaniak et al., 2014b). The second study provided contradictory results since no negative effect of chemotherapy on community diversity was found (Ma et al., 2016).

Maternal and infant infections exert a strong influence on the immunological composition of milk (Bryan et al., 2007; Riskin et al., 2012; Hassiotou et al., 2013) and, therefore, they likely impact on its microbiological composition. However, there is an almost complete absence of studies addresing this question. Human papilloma virus (HPV) infection was not correlated with a modification of the bacterial composition of milk (Tuominen et al., 2018) although the authors stated that this result might be due to the low number of HPV positive milk samples (3 out of 35) in the analyzed population.

## Transfer of Milk Bacteria to the Infant Gut

The bacteria present in colostrum and milk are among the first microbes to enter the neonatal gastrointestinal tract and, as a consequence, they may have a paramount relevance as drivers in the acquisition and development of a healthy microbiota. The role of milk as a source of bacteria, including strict anaerobes, to the infant gut has been repeteadly reported at the species and/or the strain level, by classical culture techniques (Martin et al., 2003; Martín et al., 2006; Martín et al., 2009; Solís et al., 2010; Albesharat et al., 2011; Makino et al., 2011; Martín et al., 2012b; Kozak et al., 2015; Makino et al., 2015; Murphy et al., 2017), and by culture-independent approaches (Milani et al., 2015; Asnicar et al., 2017; Duranti et al., 2017; Milani et al., 2017b). In addition, mother-to-infant transfer of *Bifidobacterium* phages through human milk has also been described (Duranti et al., 2017). However, the significance and contribution to infant gut colonization of milk bacteria remains an open question. Clinical studies trying to elucidate the question are confounded by the profound impact of non-microbial human milk components to intestinal microecology.

The microbiota of healthy breastfed infants is related to that present in the milk of their respective mothers. Pannaraj et al. (2017) suggested that approximately a quarter of the bacteria detected in infant feces during early life may derive from milk while Murphy et al. (2017) reported that the genera responsible for most of the bacterial abundance (>70%) in infant feces are shared with human milk. However, these two studies achieved genus level resolution, and only strain level analysis is valid for identifying shared taxa. Asnicar et al. (2017) described the vertical milk transmission of microbial strains, including some belonging to strict anaerobic species, and characterized their transcriptional activity once in the infant gut. Bacterial-host networks are different when breast-fed infants are compared to formula-fed infants (Martín et al., 2016), a fact that is reflected in the host transcriptome (Praveen et al., 2015). Some studies have also found long lasting differences in the fecal microbiota when comparing exclusively breastfed and non-exclusively breastfed infants (Pannaraj et al., 2017; Ho et al., 2018; Moossavi et al., 2019a). Overall, bacterial diversity and microbial pathways implied in the metabolism of carbohydrates are higher in non-exclusively breastfed infants while the contrary is observed for those pathways involved in the metabolism of vitamins, lipids and xenobiotic compounds (Ho et al., 2018).

Although the introduction of solid food during weaning was once thought to be associated with a sharp increase in the diversity of the gut microbiota in breastfed infants (Favier et al., 2002), the fecal microbiota of infants are dominated by milk bacteria, independently of the introduction or not of other foods, as long as they are breastfed (Bäckhed et al., 2015).

## Moving From Composition to Function

Human milk is a source of a wide spectrum of beneficial microorganisms that might play a role in priming the development and function of many infant systems (Ojo-Okunola et al., 2018). However, studies dealing with the functions of such microbiota/microbiome are very scarce and, as stated by Theobald Smith, (1904), “*it is what bacteria do rather than what they are that commands attention, since our interest centers in the host rather than in the parasite*”. Human colostrum and milk contain a complex array of bioactive molecules and cells, including the microbiota, which may act synergistically to preserve infant and maternal health through a wide variety of mechanisms (Morrow and Rangel, 2004).

The human milk microbiota may contribute to, at least, some of the functional properties and health benefits that epidemiological studies have associated with breastfeeding (Renfrew et al., 2012), including protection against infections, metabolic programming, immunomodulation and neuromodulation. Human milk bacteria may provide a certain degree of protection against infections caused by viruses, bacteria or fungi through a variety of mechanisms: (a) biosynthesis of compounds with antimicrobial activity, including organic acids (lactic acid, acetic acid, ethanol), bacteriocins, reuterin or hydrogen peroxide (Heikkilä and Saris, 2003; Beasley and Saris, 2004; Martín et al., 2005; Martín et al., 2006; Cárdenas et al., 2016; Cárdenas et al., 2019; Angelopoulou et al., 2020; García-Gutierrez et al., 2020); (b) coaggregation with pathobionts, impeding their access to the gut epithelial cells (Cárdenas et al., 2019); (c) competitive exclusion with pathobionts for nutrients or host receptors (Olivares et al., 2006a; Martín et al., 2010; Langa et al., 2012); (d) reinforcement of the infant gut barrier by preserving and decreasing intestinal permeability and increasing mucin biosynthesis (Olivares et al., 2006a; Vanhaecke et al., 2017; Liu et al., 2020); and (e) inmmunomodulation (Liu et al., 2020).

The analysis of the genome of some strains isolated from human milk provides some clues that may explain their anti-infectious properties. As an example, *L. salivarius* CECT 5713 is able to inhibit HIV-1 infectivity *in vitro* and its genome contains a gene encoding a protein containing a recognition motif of the high mannose N-linked oligosaccharides displayed by many pathogen antigens, such as gp120, which is essential for HIV pathogenesis (Langa et al., 2012). In developing countries, the World Health Organization (WHO) recommends exclusive breastfeeding among HIV-infected women during the first six months after birth “*unless replacement feeding is acceptable, feasible, affordable, sustainable, and safe for them and their infants*”, and to continue breastfeeding thereafter, with gradual introduction of solid foods (WHO, 2016). In these settings, the advantages of breastfeeding for mother and infant health compensate the potential risk of viral transmission (Barthel et al., 2013). Unfortunately, the contribution of the human milk microbiota in protecting from infant infections caused by this or other life-threatening viruses which are relatively frequent in developing (dengue, Ebola, and zika) or developed countries (CMV) remains largely unexplored. Interestingly, the outcome of neonatal rotavirus infections is influenced by the complex interplay between HMOs, the milk microbiome and the infant gut microbiome (Ramani et al., 2018). In the context of the ongoing pandemic caused by the SARS-CoV-2 virus, a recent study did not detect the virus in human milk (Chambers et al., 2020), and COVID-19-positive women are recommended to continue breastfeeding by the WHO and the United Nations Children’s Fund (UNICEF) (United Nations Children’s Fund, 2020).

Although most of the information about potential antimicrobial properties of human milk bacteria has arisen from *in vitro* studies and animal models, the beneficial effects of some strains have been confirmed in human clinical trials. Daily intake of a formula containing *L. fermentum* CECT5716 by 6-month-old children significantly decreased the rates of upper respiratory tract infections, gastrointestinal infections and total infections in the following 6 months (Maldonado et al., 2012). *L. salivarius* PS7, a human milk strain with a strong antimicrobial activity against several otopathogens, has been shown to be efficient in preventing recurrent acute otitis media in children (Cárdenas et al., 2019).

Studies addressing the potential role of the human milk microbiota on infant metabolism are scarce but promising. Bacterial diversity in milk is positively correlated with metabolites that are known to exert beneficial effects, including docosahexaenoic acid (DHA), as assessed by the construction of diversity-metabolites networks (Ma et al., 2016). Additionally, some species seem to be critical to regulate the concentrations of relevant milk metabolites, including inositol, DHA or butanal (Ma et al., 2016). Previous studies revealed that some strains originally isolated from human milk display metabolic activivity once in the human gut and are able to participate in the biosynthesis of functional metabolites, such as butyrate, leading to better bowel habits (Olivares et al., 2006c). More recently, administration of *L. fermentum* CECT5716 to pregnanat and lactating rats induced beneficial changes in the fatty acid composition of milk by increasing total polyunsaturated fatty acids including linoleic and α-linolenic acids and decreasing the proportion of palmitic acid (Azagra-Boronat et al., 2020).

Human milk bacteria may also be involved in immune programming of infants through different mechanisms, impacting both innate and acquired immunity (Díaz-Ropero et al., 2006; Olivares et al., 2006b; Pérez-Cano et al., 2010; Azagra-Boronat et al., 2020). Such mechanisms seem to be complementary and to exhibit a high degree of flexibility depending on factors related to the gut environment, such as the exposition to lipopolysaccharide (Díaz-Ropero et al., 2006). *L. fermentum* CECT5716 and *L. salivarius* CECT5713 behave as activators of NK, CD4+, CD8+, and regulatory T cells, and their immunomodulatory effects are different to those displayed by other strains of the same species but isolated from other sources (Pérez-Cano et al., 2010). In fact, *L. fermentum* CECT5716 is able to significantly ameliorate the inflammatory response and the intestinal damage in an animal model of intestinal inflammation (Peran et al., 2005; Peran et al., 2006; Peran et al., 2007).

As cited above, immunomodulation is involved in the anti-infectious protection conferred by some human milk bacteria or in the restoration of the damage caused by infections. *In vivo* assays have shown that *L. rhamnosus* SHA113 inhibits the expression of TNF-α and IL-6 caused by a multi-drug resistant *S. aureus* strain, restoring the concentration of leukocytes in blood (Liu et al., 2020).

Bacterial strains originally isolated from milk of healthy women are appealing as probiotic candidates because of their origin, which implies a history of safe intake by infants (Lara-Villoslada et al., 2009; Maldonado et al., 2010; Gil-Campos et al., 2012; Maldonado-Lobón et al., 2015a), and complex symbiotic interactions from the time we are born (Lara-Villoslada et al., 2007; Fernández et al., 2013; Jeurink et al., 2013). Among them, those species belonging to the genera *Bifidobacterium* and *Lactobacillus* (*B. longum*, *B. breve, L. salivarius*, *L. fermentum*, *L. reuteri*, *L. gasseri*, *L. plantarum*, and *L. rhamnosus*), have received special attention since many of them enjoy the GRAS (Generally Recognised As Safe) status (Food and Drug Administration, USA) and the QPS (Qualified Presumption of Safety) of the European Food Safety Authority (EFSA). In contrast, *S. mitis* and other mitis streptococci, *S. salivarius* and CNS have received marginal attention because of theoretical safety concerns despite of the fact that they are among the dominant bacteria both in this biological fluid (Jiménez et al., 2008a; Hunt et al., 2012; Martín et al., 2012b; Cacho et al., 2017) and in the feces of breast-fed infants (Jiménez et al., 2008a). However, they may provide relevant probiotic functions in practice (Kirjavainen et al., 2001; Uehara et al., 2001; Otto, 2009; Iwase et al., 2010; Park et al., 2011), and therefore, their potential beneficial roles in infants deserve future studies.

Desciphering the genomes of representative collections of human milk isolates and analysis of the human milk metagenome will allow the expansion of our knowledge on the functions that a “healthy” human milk microbiome should provide to the mother-infant dyad. The design of human milk bacterial consortia specifically tailored to meet early life requeriments is an attractive strategy for those infants that are devoid of the benefits of breastfeeding (Fernández et al., 2018).

## The Origins of the Human Milk Microbiota

While suckling, some oral bacteria from the mouth or nasopharynx of the infant may seed milk (Ramsey et al., 2004; Moossavi et al., 2019a); however, pre-colostrum expressed during late pregnancy contain some of the bacteria usually detected in human milk (Martín et al., 2004). In fact, oral-associated bacteria have also been isolated in precolostrum collected at the end of the first pregnancy and, therefore, before any contact with the newborn (Ruiz et al., 2014).

The origin of the microbes that constitute the oral microbiome remains widely unknown (Zaura et al., 2014) but streptococci are already present in edentulous infants (Li et al., 1997; Caufield et al., 2000; Bearfield et al., 2002; Cephas et al., 2011). The fact that these bacteria are also frequently detected in human milk (Jiménez et al., 2008a; Jiménez et al., 2008b; Hunt et al., 2011; Martín et al., 2015), might suggest a role in the acquisition or modulation of the oral bacterial communities. A recent study investigating potential relationships among bacterial communities in samples of milk, maternal and infant feces, and maternal and infant oral swabs from some mother-infant pairs found strong associations among these three complex microbial communities and *Streptococcus* was the most abundant genus not only in infant and maternal oral samples but also in milk (Williams et al., 2019). Similar results had been previously obtained by Davé et al. (2016). Biagi et al. (2017) showed that only a limited number of OTUs were shared between milk and the mouth of the infants, but they included specific *Streptococcus* and *Staphylococcus* OTUs.

**Figure 4:**
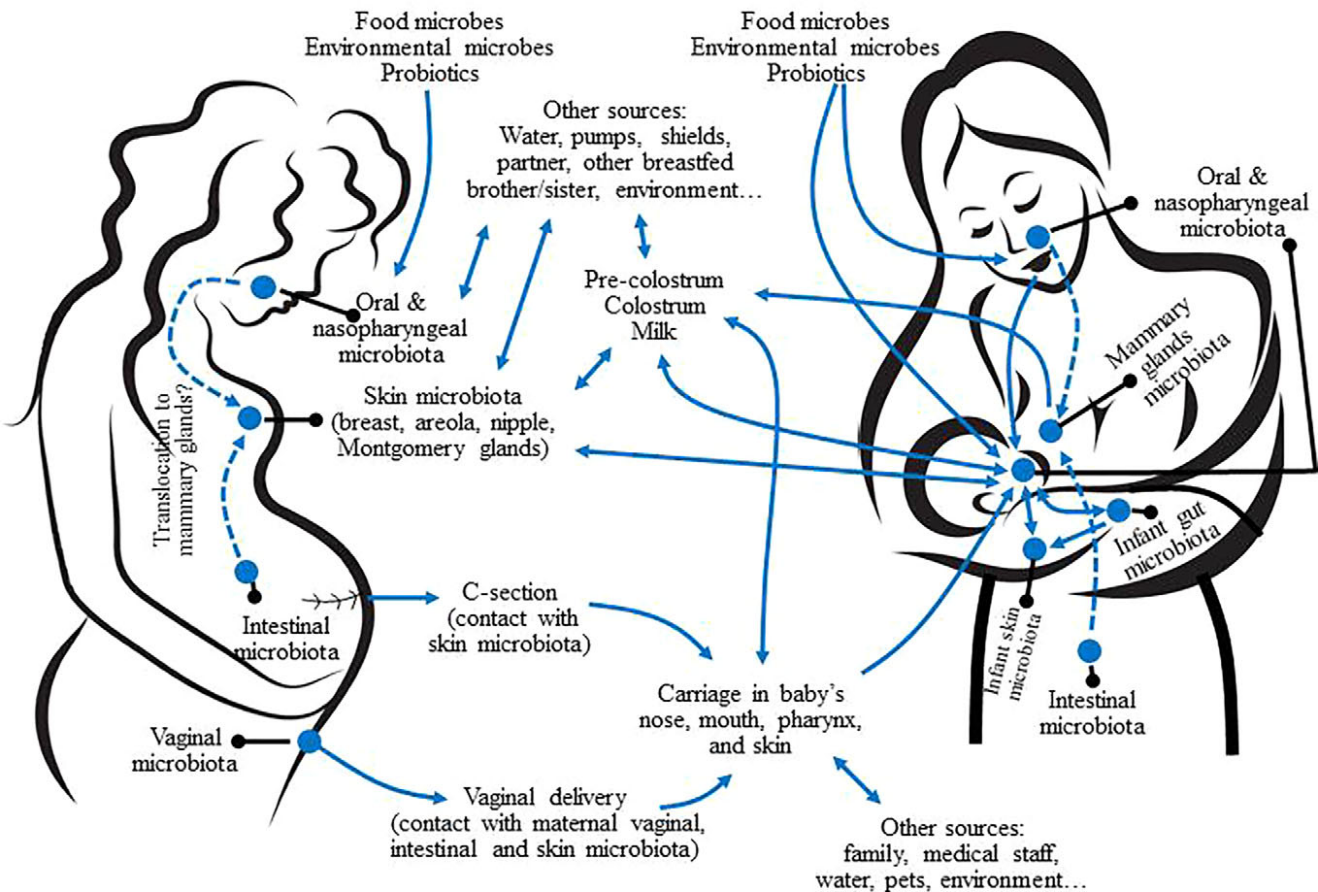
Potential sources of the microbes present in human milk and interactions with other mother-infant microbiotas. Dashed arrows represent potential translocation through an endogenous pathway.

Some species and genera commonly detected from human milk, such as *Corynebacterium C. acnes* and, especially, *S. epidermidis*, are also inhabitants of the human skin (Oh et al., 2014). Therefore, breast skin, nipples, and mammary areolas may be a source of such bacteria for human breast and milk (**Figure 4**). However, no studies on the specific transfer of skin bacterial strains to human milk or breast tissue have been performed yet. CNS, *Cutibacterium* and *Corynebacterium* are also present in all the human mucosal surfaces, including that of the digestive tract, which is, very likely, a relevant source of these bacteria for the mammary environment (**Figure 4**).

Although milk, breast skin and the infant oral cavity may share some phylotypes, there are major differences between their respective microbial communities (Hunt et al., 2011; Cabrera-Rubio et al., 2012; Jost et al., 2013; Jiménez et al., 2015). A study that compared the bacteriome of areolar skin, milk, and feces of 107 mother-infant dyads found differences in diversity and composition among the bacterial communities of the three ecosystems (Pannaraj et al., 2017).

The mucosal surfaces of the maternal digestive tract (oral cavity, gut) may be a source of bacteria for the mammary ecosystem from late pregnancy to the end of lactation (Martín et al., 2004; Rodríguez, 2014; Mira and Rodríguez, 2017; Fernández and Rodríguez, 2020). These oral- and entero-mammary routes imply a highly regulated cross-talk between bacterial cells, immune cells (dendritic cells [DCs] and macrophages) and epithelial cells. These complex cross-talks would drive the physiological translocation of certain bacteria without compromising the integrity of the gut epithelium (Vazquez-Torres et al., 1999; Rescigno et al., 2001; Macpherson and Uhr, 2004). Upon translocation, the mammary gland would exert a homing effect on the immune cells that act as bacterial carriers (Perez et al., 2007).

Low level bacterial translocation from the digestive tract to extra-digestive locations is a relatively common process (Ouwehand et al., 2001; Vankerckhoven et al., 2004; Begier et al., 2005; Dasanayake et al., 2005), while an increase in the rates of bacterial translocation from the gastrointestinal tract to the mammary environment has been observed in pregnant and lactating rodents without compromising host health (Perez et al., 2007; Treven et al., 2015; de Andrés et al., 2017; Azagra-Boronat et al., 2020). In addition, the existence of an entero-mammary traffic of immune cells during late pregnancy and lactation has long been known (Bertotto et al., 1991; Roitt, 2001; Newburg, 2005). Such efflux is responsible for the integration of the lactating breast into the mucosal immune system and its transformation in a formidable organ from the immunological point of view (Pabst, 1987; Brandtzaeg, 2010). It has also been reported that some bacterial strains isolated from human milk can cross Caco-2 monolayer by using a mechanism that involves interactions with DCs while this ability is lost in the absence of DC-like cells (Langa, 2006; Langa et al., 2012). Carrying of bacterial cells, including streptococci, lactobacilli, and bifidobacteria, by blood and milk maternal mononuclear cells has already been reported (Perez et al., 2007).

In fact, presence in milk of specific lactic acid bacteria after their *per os* administration has been reported in lactating rodents (Treven et al., 2015; de Andrés et al., 2017; Azagra-Boronat et al., 2020), lactating women (Jiménez et al., 2008c; Abrahamsson et al., 2009; Arroyo et al., 2010), or pregnant women (Fernández et al., 2016). Similarly, mothers who had ingested *L. rhamnosus* GG, *L. acidophilus* La-5, and *B. animalis* ssp. *lactis* Bb-12 can transfer such strains to their infants through breastfeeding (Avershina et al., 2018; Simpson et al., 2018).

Previously, it has been shown a *Lactobacillus* strain ingested during pregnancy could be detected in the feces of the breastfed infants, including those who were born by Caesarean section (Schultz et al., 2004). However, the authors did not investigate if the strain was present in the milk of the mothers. More recently, a *B. breve* strain detected in a rectal sample and in the milk of a woman could be also detected in the feces of her infant who was delivered *via* Caesarean section, suggesting a direct mother-to-infant transmission and supporting the possibility of a microbial translocation through an entero-mammary pathway (Kordy et al., 2020). In addition, the possibility of a shared environmental source, including the indoor microbiome, must not be ruled out.

Many transient anatomical and physiological changes may increase the translocation rate from the digestive system to the mammary glands in pregnant and lactating women (Rodríguez, 2014; Mira and Rodríguez, 2017; Fernández and Rodríguez, 2020). Such a homing effect may involve a physiological immunodepression to tolerate the fetus, a formidable angiogenesis process in the breast and the filling of the mammary duct system with pre-colostrum during late pregnancy, which would be an excellent source of nutrients for bacteria.

Although more studies are necessary to elucidate the existence of oral- and entero-mammary pathways, if this was confirmed, it would provide new approaches to manipulate the microbiota of mothers and infants inorder to improve infant health and development (Rautava et al., 2002; Martín et al., 2004).

## Mammary Dysbiosis and Lactational Mastitis

Mastitis constitutes a common feeding problem for most mammalian species and humans are not an exception (Contreras and Rodríguez, 2011). This condition is the major cause of undesired weaning from the medical point of view and represents a relevant public health issue since such premature weaning prevents the health benefits that breastfeeding provides to infants and mothers (U.S. Department of Health and Human Services, 2011; American Academy of Pediatrics, 2012; Renfrew et al., 2012).

A wide variety of bacteria may inhabit the mammary gland ecosystem during a healthy lactation period, including potential mastitis-causing species; however, if there is a disturbance of this balanced state, milk dysbiosis may occur, eventually leading to mastitis (Delgado et al., 2008; Fernández et al., 2014; Mediano et al., 2017) (**Figure 3**). Patel et al. (2017) analyzed milk samples from women suffering either subacute or acute mastitis and, also, from healthy controls. In comparison to controls, the microbiome of samples from acute and subacute cases were distinct: their diversity was drastically reduced, and they were significantly enriched in some aerotolerant bacteria, including *Staphylococcus*, while depleted in obligate anaerobes, such as *Ruminococcus*, *Faecalibacterium*, or *Eubacterium*. Similar alterations in the milk microbiome have also been found in cases of mastitis involving other mammalian species (Kuehn et al., 2013; Oikonomou et al., 2014; Derakhshani et al., 2018).

The etiology and pathogenesis of acute and subacute lactational mastitis have been reviewed by Fernández et al. (2014) and Rodríguez and Fernández (2017). Empiric use of antibiotics has been, and still is, the most common approach to treat mastitis. However, many cases do not respond to such therapy since mastitis agents are becoming increasingly resistant to antimicrobials through different mechanisms, including intrinsic resistances, presence of transmissible antibiotic resistance genes and/or the formation of biofilms (Marín et al., 2017). The high rates of antimicrobial resistance among mastitis-causing bacteria have clinical relevance in relation to treatment options. In addition, wide-spectrum antibiotics may alter the bacterial composition of milk, impairing vertical transmission of microbes through breastfeeding (Soto et al., 2014). Therefore, new strategies for the management of mastitis are needed, and in this context, those based in the modulation of the mammary bacterial communities through the selection and application of probiotic strains that were originally isolated from human milk seem particularly suited for this target (Fernández et al., 2014).

Several human trials have shown that oral administration of some human milk strains (*L. salivarius* CECT5713, *L. salivarius* PS2, *Lactobacillus* *gasseri* CECT5714, *L. fermentum* CECT5716) provoke relevant changes in a variety of milk microbiological, biochemical and immunological parameters, including a significant decrease in the concentration of mastitis-causing agents (Jiménez et al., 2008c; Maldonado-Lobón et al., 2015b; Espinosa-Martos et al., 2016). In fact, such an approach has been found to be more efficient than empiric antibiotherapy for the treatment of this condition (Arroyo et al., 2010). Metabolomic studies have revealed that the impact of the probiotic treatment can be also observed in the urine of the treated women (Vazquez-Fresno et al., 2014). As an example, lactose was present in urine samples before the treatment but it was no longer detected after the probiotic treatment, indicating a restoration of the integrity of the mammary epithelium. Assessment of transcriptomic changes in milk somatic cells associated with the intake of *L. salivarius* PS2 by women with mastitis has also provided valuable information about the potential mechanisms responsible for the efficacy of specific probiotics in treating mastitis (de Andrés et al., 2018). Finally, a few strains (*L. salivarius* PS2 and *L. fermentum* CECT5716) have been successfully applied as a preventive strategy compared to a placebo, when administered either during late pregnancy (Fernández et al., 2016) or during lactation (Hurtado et al., 2017) to women with a history of mastitis after previous pregnancies.

Significant increases in the milk concentrations of TGF-β2 and IgA have been observed after intake of other probiotics during pregnancy or lactation (Rautava et al., 2002; Prescott et al., 2008; Nikniaz et al., 2013), suggesting that the probiotic approach may control the growth of mastitis-causing bacteria in the mammary gland preventing potential damage to the mammary epithelium.

## Microbiota of the Breast Tissue of Non-Lactating Women

Most microbial studies addressing the human mammary ecosystem have been focused on the lactation period and limited to the analysis of colostrum and milk samples. However, the number of studies dealing with the microbiota of breast tissue in non-lactating women is rapidly increasing, particularly in the frame of breast cancer research. Breast cancer ranks as the most common malignancy in women. Some studies have observed a correlation between dysbiosis of the gut microbiota and breast cancer (Goedert et al., 2015; Goedert et al., 2018; Laborda-Illanes et al., 2020). In addition, changes in the microbiota of breast tissue have also been linked to breast cancer.

In a pioneering study, samples of breast tissue from 81 women with and without cancer were analyzed by sequencing of the V6 region of the 16S rRNA gene (Urbaniak et al., 2014a). Sequences corresponding to *Staphylococcus*, *Cutibacterium*, *Acinetobacter*, *Enterobacteriaceae*, *Bacillus*, *Pseudomonas*, or *Prevotella* were detected in a high percentage of the samples, including some obtained from women without a previous history of lactation. Cultures confirmed the presence of viable bacteria in some of the samples. In parallel, Xuan et al. (2014) employed 454 pyrosequencing of the V4 region of 16S rRNA gene to analyze breast tissue samples from 20 patients with breast cancer, each one providing tumor tissue and normal adjacent tissue. These authors found that *Methylobacterium radiotolerans* was enriched in tumor tissue, while *Sphingomonas yanoikuyae* was enriched in normal tissue. However, some doubts arise from their results since this technique does not discriminate at the species level, the DNA of the two genera that discriminated between both groups are typically found in DNA extraction reagents and kits, and no blank controls were included in the study. Later, Urbaniak et al. (2016b) reported that the bacteriome of breast tissue adjacent to breast cancer differs from that found in breast tissue from healthy controls undergoing cosmetic surgery. Compared to healthy controls, the relative abundances of *Bacillus*, *Enterobacteriaceae* and *Staphylococcus* were higher in samples from women with breast cancer while the abundance of lactic acid bacteria was lower (Urbaniak et al., 2016b).

Hieken et al. (2016) found different bacterial communities in breast tissue from women with breast cancer in comparison to women suffering from a benign breast disease. Malignancy was associated with an increase in some taxa that were present at a low abundance level, including the genera *Atopobium*, *Fusobacterium*, *Gluconacetobacter* and *Hydrogenophaga*. Chan et al. (2016) investigated the presence of bacteria in the nipple aspirate fluid obtained from women with a previous history of breast cancer and from healthy women and found a higher incidence of the genus *Alistipes* in the first group. In addition, the bacteria associated with breast cancer shared β-glucuronidase activity and, therefore, the authors suggested that this enzymatic activity may promote breast cancer. Recently, two studies described racial differences in the microbiome of breast tumors (Smith et al., 2019; Thyagarajan et al., 2020) while another study identified differences in diversity and composition not only between tumor and normal tissue but also among women and between the breasts of the same woman (Klann et al., 2020).

The microbiota may promote or inhibit tumorigenesis by several mechanisms, including the alteration of immune responses or the effect of bacterial-derived enzymes (e.g., β-glucuronidase and β-glucosidase activities) and metabolites, such as short-chain fatty acids, lipopolysaccharides, secondary bile acids, estrogens, or genotoxins (Kovács et al., 2020). In addition, the microbiota and its metabolites may exert a strong influence on the efficacy of chemotherapy and radiation therapies (Kovács et al., 2020). Overall, the results of previous studies warrant further research to elucidate the relationships between breast microbiota and breast cancer.

The breast microbiome may also play a key role in the outcomes of breast plastic or cosmetic surgery, including breast reconstruction, breast reduction or breast augmentation. Breast augmentation is one of the most frequent cosmetic surgical procedures practiced worldwide and can lead to several complications, capsular contracture being the most common one (Rieger et al., 2013; Cook et al., 2020). Bacterial growth, and subsequent biofilm formation, is one of the main risk factors for capsular contracture (Dancey et al., 2012; Ajdic et al., 2016; Walker et al., 2019). It has been suggested that chronic biofilm infection of breast implants may be implicated in the development of breast implant-associated lymphoma (Hu et al., 2015; Hu et al., 2016). Bacteria that are often associated with human milk, breast tissue and breast skin (*S. epidermidis* and other CNS species; *C. acnes*) have been repeatedly isolated within or surrounding breast implants from patients with capsular contracture by using classic culture-based approaches (Virden et al., 1992; Dobke et al., 1995; Ahn et al., 1996; Pajkos et al., 2003; Galdiero et al., 2018) and culture-independent techniques (Cook et al., 2020). At present, the origin of the bacteria detected in the explants (breast tissue and skin contamination) remains unclear (Bachour et al., 2019), and more studies are required to elucidate the role of mammary-related bacteria in the tolerance toward these devices or in the adverse outcomes that are relatively frequently associated with their implantation.

## Future Trends and Conclusions

Conflicting results when trying to analyze the factors that may play a role in shaping the composition of the human milk microbiota can be explained, at least partially, by the low number of samples/women analyzed in most of the studies carried out so far and, also, by many host, environmental, perinatal, and technical factors (LeMay-Nedjelski et al., 2018). International and collaborative studies, involving a high number of participants and performed under identical conditions, are required in order to elucidate the impact of these factors and their interactions.

The origin of the microbes found in the mammary ecosystem remains a largely open question. Some of the bacteria detected in milk most likely originate from the maternal skin and areola. During lactation, shared features between the microecology in the infant mouth and milk suggest interactions but the precise directions and significance of which are yet to be determined. Perhaps most intriguingly, experimental and human data indicates that some bacteria in milk may originate in the maternal digestive tract and that an enteromammary pathway for microbes may exist. Further assessment of this hypothesis demands sophisticated experimental and clinical studies and state-of-the-art methods to ensure accurate strain-level identification of specific bacteria in not only the intestine and milk but also in the bloodstream and within the immune cells thought to be responsible for the transfer.

The emerging data indicating that various maternal characteristics and exposures, including BMI, antibiotic exposure, gestational age or delivery mode, are associated with the composition of the milk microbiota suggest that the microbes in milk are linked to health and disease in the mother and perhaps, also, in the infant. As of present, however, the biological role and clinical significance of the bacteria in human milk remain poorly characterized and several fundamental questions require elucidation. Detailed metagenomic, metatranscriptomic and metabolomic studies are paramount for understanding the functionality of the milk microbiota. It is also important to appreciate the fact that most published studies describing the bacterial communities of human milk are based on culture-independent molecular methods, such as qPCR and sequencing of either of the 16S rRNA gene or the whole bacterial genome. While these tools are highly sensitive, detection of bacterial DNA does not entail the presence of viable or even intact bacteria. Furthermore, there are published data indicating that at least some bacteria in milk may actually be found on and inside immune cells (Perez et al., 2007). Distinguishing between intact and viable free or intracellular bacteria and the mere presence of bacterial fragments is a crucial step in understanding their biological function. The presence of bacterial components such as DNA in milk may be sufficient to induce immune responses in the mammary epithelium, immune cells, or the neonatal gut. Milk immune cells interacting with bacteria, on the other hand, may mediate immune responses specific to these bacteria. Given the role of human milk in establishing immune tolerance towards antigens in the maternal diet (Verhasselt et al., 2008), it is conceivable that milk may serve as a vehicle for inducing tolerance in the newborn to colonizing microbes from the mother. Currently, no direct evidence to corroborate or refute this hypothesis exists.

Live bacteria in human milk serve biological purposes in the mother. The relationships between the bacterial composition of milk and the risk of mastitis suggest that the indigenous bacteria may be necessary for mammary gland health. Mammary bacteria may also play some roles in the non-lactating mammary gland and the development of breast carcinoma, which is a subject of substantial clinical significance and an area of active research.

In addition to implications to maternal health, the human milk microbiota may be transferred to the infant, potentially acting as a driver in early oral and gut colonization. As reviewed above, shared bacterial taxa detected in maternal stool, human milk and the infant gut suggest that milk may be a vehicle for early colonization. This has to be interpreted with caution since there is the possibility of other routes of bacterial transfer and taxonomic discrimination should be performed at the strain level to confirm identity. As in the case of the origin of the milk bacteria, experimental studies in animal models offer a more reductionist means of dissecting the role of milk microbes for gut colonization. A translational approach complementing clinical studies with basic science is therefore needed.

So far, most studies dealing with the human milk microbiome have been focused on the taxonomical composition and only a few of them have dealt with its potential functions for the infant-mother dyad. Breastfeeding has been associated with reduced risk of chronic conditions such as obesity (Victora et al., 2016), which has also been linked to aberrant early gut colonization. The extent to which the beneficial health effects of breastfeeding are mediated by modulation of the developing gut microbiota remains an open question. Even less clear is the impact of human milk bacteria on child health. Indeed, studying the roles of the human milk microbiota in health and disease is a difficult task since there are synergistic activities among different milk molecules and cells, and several non-microbial components in human milk have the potential to modify the infant gut microbiota. However, we have a rapidly increasing variety of powerful *in vitro* and *in vivo* tools, techniques, and procedures to address such a question from cell biology to well-designed clinical trials, from human breast organoids to human milk microbiota-associated mouse models (Wang et al., 2017) and from true metagenome (integrating data from microbiome, microbial genomes and human genome projects) to systems biology approaches.

## Author Contributions

All the authors contributed equally to the review of the literature and to the writing and revision of the manuscript. All authors contributed to the article and approved the submitted version.

## Funding

PSP receives grant support from R01 HD100542-01. JMR and LF are recipients of grant PID2019-105606RB-I00 (Ministerio de Ciencia e Innovación, Spain).

## Conflict of Interest

The authors declare that the research was conducted in the absence of any commercial or financial relationships that could be construed as a potential conflict of interest.
